# Examining preferences for weight loss interventions among black men and women with overweight or obesity: A qualitative study

**DOI:** 10.1186/s12889-025-24144-7

**Published:** 2025-08-16

**Authors:** Snehaa Ray, Christie I. Idiong, Rachel Anderson, Kate Killion, Curtis Antrum, Michael Puglisi, Jolaade Kalinowski, Kristen Cooksey Stowers, Loneke T. Blackman Carr

**Affiliations:** 1 Department of Nutritional Sciences, University of Connecticut, 27 Manter Road, Unit 4017, CT 06269-4017 Storrs, USA; 2 Department of Allied Health Sciences, University of Connecticut, Storrs, CT USA; 3 Department of Human Development and Family Sciences, University of Connecticut, Storrs, CT USA

**Keywords:** Obesity, Weight loss, Black adults, Health disparity

## Abstract

**Background:**

Behavioral interventions aiming to modify dietary habits and physical activity have been less effective in achieving clinically significant weight loss in Black adults. Inequities exist in both representation and weight loss outcomes in among Black men and women compared to White men and women. While there have been some research efforts focused on weight loss in Black women, participation rates of Black men in weight loss interventions are lower. This may perpetuate the development of obesity-related conditions such as diabetes, heart disease, and other cardiovascular problems. This qualitative study investigated the barriers and facilitators to healthy eating and physical activity and the weight loss desires among Black adults with overweight or obesity.

**Methods:**

24 Black adults mainly from New England states were recruited for the study. Semi-structured interviews were conducted with Black males and females who self-reported being overweight or obese. Preferences for weight loss specific to each gender, barriers, and facilitators for weight-related behaviors such as diet and physical activity, were examined by gender to inform development of a culturally relevant behavioral weight loss intervention.

**Results:**

The sample consisted of Black adults from diverse racial and ethnic backgrounds. Black females (*n* = 16) and Black males (*n* = 8) were mostly non-Hispanic Black Americans. Key themes that emerged were: (1) the creation of a weight loss program for Black adults, (2) healthy eating barriers, (3) physical activity barriers, (4) healthy living facilitators, and (5) social support. Distinctive preferences for weight loss were expressed by Black females and males Community and personalization were preferred by Black females while Black males preferred personalized diet and exercise regimes for diverse health conditions, not limited to weight loss. While both genders referred to digital devices and apps for recording weight, diet and physical activity as a major facilitator to adopting healthy habits, social support in the form of culturally relevant information from healthcare providers was highly desired to be incorporated into the intervention.

**Conclusion:**

The findings of this study hold relevance for designing and developing of weight loss programs that promote behavior change for Black adults and help reduce obesity-related health inequities within this population.

## Introduction

Obesity remains a persistent public health challenge, impacting 42.0% of adults in the United States [1]. Black adults are disproportionately affected with nearly 50.0% living with obesity, a prevalence that is greater than other racial and ethnic groups [2]. The disproportionate burden of obesity increases Black adults’ risk of developing preventable diseases such as type 2 diabetes, cardiovascular diseases and some types of cancers [3].

Efficacious obesity treatment is found through behavioral weight loss interventions [4, 5]. Seminal trials combine cognitive behavioral therapy, dietary modification, and physical activity behavior change to produce clinically meaningful weight loss of 5–10% [6, 7]. While behavioral weight loss interventions are regarded as efficacious, consistent challenges exist concerning treatment among Black adults [8]. In seminal weight control trials, Black men remain underrepresented precluding them from experiencing the benefits of obesity treatment [9–11]. When Black men are engaged in behavioral weight loss interventions, positive and clinically meaningful outcomes have resulted [8]. However, a disparity between Black and White men’s weight loss exists, with approximately 2% lower weight changes observed [10, 11]. A similar inequity in weight loss has been observed between Black and White women, with White women typically achieving greater weight loss from baseline compared to Black women [12]. Notably, among Black women, weight losses produced through standard behavioral weight loss treatment interventions are typically modest (2–3%) and below the clinically meaningful target of 5–10% [13, 14]. While behavioral weight loss interventions for obesity treatment have been established as the standard of care, disparities in representation and outcomes for Black adults reflect a need to reconsider what may and may not work for this population subgroup [8, 15]. Thus, there is a need to determine the best approach to engage Black adults and to develop weight loss interventions that can consistently produce clinically meaningful outcomes to improve their health.

Current evidence indicates a need to understand weight-related behaviors such as diet, physical activity and what supports or creates barriers to the adoption of these behaviors [13, 16, 17]. While quantitative outcomes have illustrated the need for continued development of weight loss interventions, investigators have called for the use of qualitative methods to understand disparities in treatment and engagement among Black adults [8, 17, 18]. Qualitative research provides valuable insights into lived experiences, contextualizes quantitative outcomes, and informs intervention design to better address obesity treatment in Black adults [12, 19]. Using qualitative methods, this study examined the barriers and facilitators of behavioral targets of weight loss interventions (healthy diet, physical activity), and identified desires for weight loss treatment among Black adults with overweight or obesity.

## Methods

### Research Team

To maintain credibility in research, a diverse group of researchers from interdisciplinary backgrounds were involved in the study. This qualitative study was developed by four researchers (KCS, LBC, MP, and JK) with a focus on health equity research. A five-person team comprising 4 females and 1 male (henceforth referred to as the working team) led the entire research process. The working team, was guided through the process of data collection and data analysis by Co-principal investigators (Co-PI) KCS and LBC with expertise in research aimed at reducing obesity-related health disparities. The semi-structured interview guide was developed by principal investigators LBC and KCS. LBC and a doctoral-level researcher (SR) with a background in nutritional sciences and qualitative coding conducted the interviews. The qualitative analysis process was collaboratively managed, and Co-PI KCS led the analysis. Co-PI KCS and two trained graduate assistants (KK and RA) drafted the initial codebook. Co-PI LBC contributed significantly by addressing discrepancies in the codebook, and supporting the generation of themes. Trained graduate level coders (CI, KK, RA, and CA) with a background in allied health contributed to qualitative coding and theme generation. The final themes were agreed upon by the full research team. Two trained graduate research assistants (KK and RA) performed data management and ran the analysis. The manuscript was drafted by SR, CI, and LBC, with KK, RA, CA, MP, and KCS contributing to the review process.

### Recruitment

Active recruitment occurred in collaboration with community partners from a mid-sized New England city just before and during COVID-19. They initiated communication with individuals interested in the study, as well as organizations from which interested individuals may be potentially recruited. Individuals interested in the study were then contacted by the study team. Male recruiters within the community partner team specifically engaged with potential Black male participants to encourage their interest in joining the research study. Due to the emergence of COVID-19, in-person research activities were halted, and recruitment strategies were expanded to include individuals beyond the initially targeted local region. Passive strategies were also implemented during recruitment. This included the use of ResearchMatch, a nationwide online recruitment resource for health research, supported by the National Institute of Health [20]. In the local community, additional strategies for recruitment included the distribution of quarter cards, flyers, and listserv email announcements.

### Eligibility criteria

Individuals were considered eligible for the study if they self-identified as either Black, African American, or Afro-Caribbean, had a body mass index (BMI) ≥ 25 kg/m^2^, based on self-reported height and weight, and expressed a willingness to participate in audio-recorded individual interviews. Individuals who expressed interest in the study were contacted via telephone by a study staff member. If no response was received, then the participants were contacted through email requesting a call back to describe the study in detail to determine their interest and eligibility. After an individual expressed interest and confirmed a date and time, the virtual interview was scheduled.

### Data collection

Individual interviews were conducted between August 2020 to August 2021 using a semi-structured interview guide. The interview guide was developed based on the scholarly literature indicating the gaps in knowledge and challenges regarding weight loss and behavioral obesity treatment with Black adults [12, 19, 21]. Table 1 provides a list of questions that were asked during the interview. Current evidence indicated a need to understand weight-related behaviors such as diet, physical activity and what supports or creates barriers to adoption of these behaviors, therefore the interview guide was broken into sections with probes pertinent to each question. The WebEx software was used to conduct and audio record the interviews. Prior to the administration of the interview questions, informed consent was gained from participants. A doctoral-level trained moderator conducted the individual interviews. A note taker was present to record any verbal and non-verbal communications in the event that there was a recording malfunction or internet disconnection. Each interview lasted about 35–45 min. At the end of the interview, participants were asked to complete a short survey to collect demographic information. Participants were provided a $50 gift card for their participation in the study.

**Table 1:** Sample semi-structured interview questions

Interview Questions	Probes
Section: Feedback on intervention components 1. What are your first thoughts about what’s included in a typical weight loss program? 2. What do you think a weight loss program for Black adults should look like? 3. What is missing in the typical weight loss program for Black men/women? (asked according to self-identified gender) Section: Barriers 4. What gets in the way, or makes it hard, for you to eat healthy? 5. Which healthy eating barriers are the hardest for you to overcome? 6. Which healthy eating barriers are the easiest to work around? 7. What barriers, or things get in the way, of you getting regular exercise? Section: Assets 8. What makes it possible, or easier, to eat healthy? 9. What makes it possible, or easier, to be active? Section: Social Support 10. What are the ways others support you to eat healthy foods? 11. How do people support you to be physically active (Eg. exercise) in your spare time?	• What should be added, or removed, for Black men/women (ask according to self-identified gender) • If you were in a weight loss program for Black adults, what do you want to have included? • Cooking skills • Flavor/seasoning food • Taste preferences • Time • Recipes • Food Storage information • Transportation • Cost • Time • Opportunities to sit • Safety • Family commitments • Hair care • Home resources • Community resources • Educational • Religious structures/faith-based (ex. Church, Mosque) • Community programs • Work (people, places, structures) • Technology (ex. Phone apps, websites, etc.) • Who gives you support? (family, friends, coworkers) • How is the support different between people?

### Demographic Information

Participants completed a demographic survey including information such as age, gender, BMI, racial heritage, Hispanic or Latino ethnicity, ancestry, education, marital status, household income, and employment status. Categorical variables were represented through frequencies and percentages while continuous variables were summarized using means and standard deviations. The SPSS software version 29.0 was used to analyze all quantitative data.

### Data analysis

**Fig. 1:**
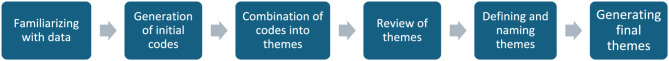
Six-Step Thematic Analysis Process

Qualitative data were audio recorded and transcribed verbatim. A team-based approach was used to code individual transcripts and using Dedoose software version 4.12. The thematic analysis, consisting of a 6-step process developed by Braun and Clarke (Fig. 1), was followed to analyze data and generate themes [22]. To increase familiarity with the transcripts 3 members of the coding team (KCS, KK and RA) listened to audio recordings and read the transcripts several times to develop and revise the coding guide after applying the coding scheme to a subsample of transcripts. Then the larger research working team, consisting of the co-PI (KCS) and 3 pairs of trained graduate students met to compare codes and provide code definitions. Through an iterative process, the team and principal investigator convened to refine and finalize the coding guide (comprising codes and definitions). With the finalized codebook, 3 pairs of coders coded all transcripts (individually and then met to reconcile differences) and convened to discuss the analysis and to generate the final themes. The coders achieved an average inter-reliability score of 0.83.

## Results

In the current study, twenty-four Black adults who closely represent the Black population of Connecticut participated in this study with an average age of 46.0 ± 13.3 years and were predominantly female (*n* = 16; 66.0%). The mean BMI of the total sample was 33.0 ± 8.0 kg/m2, with half of the participants classified as overweight and half having obesity. The majority of participants identified as Black American (75.0%, *n* = 18), with 20.8% (*n* = 5) of Afro-Caribbeans. Most were non-Hispanic (91.0%, *n* = 22), born in the United States (75.0%, *n* = 18), and residing in Hartford, Connecticut (46.0%, *n* = 11). Over half reported a household income exceeding $60,000 (59.0%, *n* = 14), and 29.0% (*n* = 7) had some college education. The description of the sample is provided in Table 2.

**Table 2:** Baseline demographic variable of study participants

Variable	*N*	(%)
Sex		
Female Male	16 8	66.7 33.3
Age (Mean [SD])	46.1	13.3
Weight Status		
Overweight Obesity	12 12	50.0 50.0
Location		
Connecticut Other States	11 13	45.8 54.2
Racial Heritage		
African American Afro-Caribbean American Indian/Alaska Native	18 5 1	75.0 20.8 4.2
Hispanic/Latino Ethnicity		
Non-Hispanic Hispanic/Latino	22 2	91.7 8.3
Household Income		
>$60,000 $40,000-$60,000 $10,000-$30,000 <$10,000	10 5 4 3	41.7 20.8 16.7 12.5
Immigration Status		
Born in the US Not born in the US	18 6	75.0 25.0
Marital Status		
Single Married Divorced Widowed	13 5 4 2	54.2 20.8 16.7 8.3
Highest Education		
Graduate School or Professional Education College/University Degree Some College Education (< 4 years) Technical School or Vocational Training Graduated from High School Did not complete high school	5 7 7 1 3 1	20.8 29.1 29.1 4.16 12.5 4.1

The following themes were generated from the interviews with Black adults who were either overweight or obese. A summary of the key themes, subthemes, and representative statements of the Black female and male participants are presented in Table 3 (females) and Table 4 (males).

### Theme 1: Weight loss program preferences

Participants expressed their preferences regarding how a weight loss intervention could be made more desirable to Black adults. Black female participants indicated incorporation of the local community, and group activities and to model community programs that are successful. They stressed the importance of community, as participants identified that the community was where they could source support, motivation and engagement. Black female participants desired personalization in weight loss interventions through the incorporation of flavorful and culturally relevant recipes tailored to the preferences of Black adults and personalized exercise routines and avoiding general prescriptions for diet consumption and exercise engagement.

Black male participants, on the other hand, expressed a strong interest in a program that was specifically customized for Black adult males, emphasizing healthy eating and exercise. They highlighted the desire for awareness and education concerning certain health conditions (e.g. hypertension, diabetes, and arthritis) as well as promoting tailored recipes designed to manage health conditions such as low sodium and diabetes-friendly recipes.

Both genders highlighted the importance of a personalized program that includes flavorful and culturally relevant recipes tailored to the preferences of Black adults. A preference for recipes that reflect the cultural traditions of Black adults while promoting healthier adaptations of traditional food were expressed such as recipes that incorporate ingredients and cooking methods rooted in African and Caribbean heritage. While Black female participants predominantly emphasized community as a necessity in weight loss programs, Black male participants emphasized the need for a program that will concentrate not just concentrating on weight loss but also connects to other health concerns such as type 2 diabetes and hypertension.

### Theme 2: Healthy Eating Barriers

The primary barrier included a lack of knowledge and skills in preparing nutritious meals, scarcity of healthy recipes, and general dislike for cooking. Specifically, female participants indicated limited or no time as the primary barrier to cooking healthy meals. They also emphasized family responsibilities as a significant challenge to cooking and consuming healthy foods. Male participants identified time constraints for cooking healthy meals, grocery shopping, and family commitments as significant barriers to consuming healthy meals. Both male and female participants frequently cited time constraints and family responsibilities as primary barriers to consuming healthy meals.

### Theme 3: Physical Activity Barriers

Black female participants frequently discussed a lack of motivation to exercise and challenges in finding the time to maintain an exercise routine as the primary barriers to engaging in physical activity. Black female participants uniquely mentioned that time constraints were mainly due to career and job commitments and family responsibilities. Black male participants identified time as the primary barrier to physical activity. Additionally, they mentioned lacking motivation to create and maintain an exercise regime. Both genders indicated a lack of time and motivation as the primary barriers to engaging in physical activity. Both genders reported similar barriers to engaging in physical activity, with no variation in their experiences.

### Theme 4: Healthy Living Facilitators

Technology, mindset and routine were emphasized to have a high influence for engaging in healthy eating and physical activity. Female participants indicated an openness to engaging with technology-delivered resources and guidance for nutrition and exercise. This included smartphone apps such as MyFitnessPal and social networking sites, such as Facebook. Responses stated by female participants highlighted mindset and routine, defined as engaging in health behaviors out of routine and keeping motivated towards those healthy behaviors as major factors to facilitating healthy lifestyle choices. These two factors were cited as driving forces to maintain motivation to engage in healthy behaviors such as healthy eating and physical activity. Black female participants emphasized the inclusion of exercise rooted in their culture, such as dance-based workouts like Zumba or hip-hop. They also highlighted the value of walking and running groups which foster a strong sense of community. Male participants highlighted having access to online resources to find information and guidance on healthy recipes, exercise and social media applications that create an online community. Male participants noted routine as a necessity for engaging in healthy behaviors. Specifically, initiating and maintaining an exercise routine was highlighted as the primary motivation for engaging in physical activity.

Mindset and routine were emphasized equally among male participants compared to female participants. The notable gender contrast was highlighted among facilitators, with female participants predominantly expressing a desire for technology to engage in healthy behaviors whereas male participants sought online resources and information for managing weight and other health conditions.

### Theme 5: Social Support

Participants’ perceptions about how they felt best supported to engage in healthy behaviors varied by sex. Female participants mentioned feeling most supported by healthcare professionals and the advice for selecting health behaviors regarding nutrition and physical activity. Coworkers were also mentioned by female participants as sources of support for engaging in healthy behaviors. Female participants specifically noted feeling most supported when engaging in healthy behaviors with family members and receiving verbal encouragement from a person in their social network.

Male participants noted support from healthcare professionals and coworkers to be a trusted source when looking to engage in eating healthy and exercise but ranked their friends higher when considering who influenced engaging in healthy behaviors most. Examples specifically noted included sharing meals or walking together as large facilitators for eating healthy and engaging in exercise, respectively. Both female and male participants found the least support from romantic partners.

**Table 3:** Summary of themes and representative statements among female participants (*n* = 16)

Key Themes	Subtheme (code frequency *n*)	Representative Statements
Creating Weight Loss Program for Black Adults	Community consisting of like-minded people for support and motivation (*n* = 54)	“Like, um, you know, um, and that’s not always like directly related, but to be honest, any type of support, or just like an, an internal, like community building that you can do with Black people will, will help any program, even if it’s not directly related to weight loss”, *Age 26*
	Personalization to meet individual preferences (*n* = 54)	“Yeah, definitely, because I feel like each person gets excited about a different thing, like not everyone- You know, a goal about how they… Why fitness is important. So, um, yeah, personalization is definitely important, in my opinion.” *Age 40*
Healthy Eating Barriers	Lack of time for healthy eating (*n* = 47)	“Because you have to it’s… When you don’t know, or you trying to learn something, it takes time. When it’s something effortlessly that comes together. Like if you were to ask me to make, um, fried plantains, I could do that within seconds. But if you were to ask me a big potato or something in the oven, I had to sit down and I have to Google it, look for recipes and it takes a lot more time.” *Age 29*
	Lacking cooking knowledge and skills(*n* = 17)	“Um, so, and then with cooking too, sometimes, like, the- the details to, like, paying attention to this, how long you have to leave this, or you’re trying to cook multiple things at once, something might get cold.” *Age 45*
Physical Activity Barriers	Lacking of motivation for exercise (*n* = 44)	“Uh, what makes it hard? Um, sometimes I just don’t have the energy, sometimes, um, sometimes I’m just, just tired, uh, so you know, that does it.” *Age 26*
	Lack of time for exercise(*n* = 36)	“Just not getting up and building the extra time into your schedule. Well, it [time] can be affected by work if I am traveling and my day may start at 8:00 AM, and I might not get home till 10:00 PM. So I know if I’m having a long day, I might not get up that extra hour that morning and go workout because I know I have a eight hour work day, then I have six more hours of travel.” *Age 44*
Healthy Living Facilitators	Mindset and routine for maintaining healthy behaviors (*n* = 107)	“It’s just a mindset. You make a mind, where you tell yourself, “I gotta do this. I gotta walk.” For example, exercising, I gotta go down the street now, and you walk till you feel tired or about to get tired, and you come right back. So, it’s just a whole mindset, ‘cause I have to wake up before six, or by six in the morning. And, it’s a whole mindset, if I have to do something, I have it do it. Nothing stops.” *Age 53*
	Using technology in the form of online tools to engage in healthy behavior (*n* = 84)	“I mean really what motivates me is just really just social media. I go on YouTube. YouTube is like is life. I go on YouTube every day and I’m pretty proud to say that because YouTube for me is like, I don’t go in there just to watch other people’s life. I go on there and I learn so much, like I have, you know, I could list like five people right now who are like influencers who take really good pride, like in, in their content. And they talk about like their diet and what they eat. And they also upload like exercising videos, you know, some of them are really… So I get a lot of inspiration from just like looking at their bodies and I’m like, oh wow. Like I know I can get to that.” *Age 26*
Social Support	Family (*n* = 86) and Friends (*n* = 76) encouraging to eat healthy and exercise	“And the receive, the support I received from my mom It’s more, it’s more, um, knowledge in anything else. So I guess everybody plays a different part in it and it’s in a way a compliment and it builds the whole pyramid.” *Age 29*
	Social support beyond family and friends (*n* = 92)	“Yes, absolutely. I have some coworkers that are very health conscious. So we are often just talking about like, who’s the health person in the office. They’ll recommend that we try or eat and that’s type of thing.” *Age 37*

**Table 4:** Summary of themes and representative statements among male participants (*n* = 8)

Key Themes	Subtheme (code frequency *n*)	Representative Statements
Creating Weight Loss Program for Black Adults	Community consisting of like-minded people for support and motivation (*n* = 32)	“Um, I mean, I think that all sounds good. I do, I think the group component again is a big part of success when you’re able to communicate with other people going through the same program. Um, and then again, the healthy choice, healthy options for food” *Age 19*
	Personalization to meet individual preferences (*n* = 40)	“Um, I would, like, I feel like the like I don’t know, about like the exercise in particular. I feel like, I feel like, it depends on the person, you know? I feel like it depends on the person.” *Age 32*
Healthy Eating Barriers	Lack of time for healthy eating (*n* = 37)	“So obviously the time commitment, uh, if I’m not meal prepping, finding, it’s easy to go to a fast food restaurant and get, you know, the popular meals that you like, or, um, you know, just getting something quick. the amount of time it takes to prepare meal versus going across the street and buying something, I think, um, you know, obviously it’s less amount of time to purchase something that’s already prepared.” *Age 32*
	Lacking cooking knowledge and skills (*n* = 33)	“I don’t cook, so. And I wanna learn but I haven’t uh, I haven’t taken the steps to learn yet.” *Age 65*
Physical Activity Barriers	Lacking motivation for exercise (*n* = 20)	“I think that- that is the biggest one, outside of, um, I guess, um- um, motivation. Sometimes you- you won’t always be motivated to do it.” *Age 39*
	Lack of time for exercise (*n* = 24)	“Well, 30 minutes a day is way too much time. (laughing) No one has time for that. Um, I’m sure that if I went jogging every morning, or went to the gym every day, I would probably be healthier but, you know, big time factor. I mean, no one has time for that, you know.” *Age54*
Healthy Living Facilitators	Mindset and routine for maintaining healthy behaviors (*n* = 49)	“I would say the main thing for me [that makes it easier to be active] is maintaining a routine. It’s so much easier when you’re accustomed to doing things, you know.” *Age 54*
	Using technology in the form of online tools to engage in healthy behavior (*n* = 56)	“I have an Apple watch and several of my friends do too. I guess this isn’t, us talking, but we are communicating through like … I guess this is more of an encouragement … If you see someone close their activity rings, or they, had a workout, that is sometimes encouraging to get those messages in.” *Age 36*
Social Support	Family (*n* = 36) and Friends (*n* = 47) encouraging to eat healthy and exercise	“They [friends] know that I’m, I’m trying to uh, s- be active. So they, they uh, I have a few friends who’ll come by and try to get me to go out and do things… if you wanna call it exercise, yeah, some of ‘em do. Some of ‘em will take a walk every now and then but usually it’s just me by myself.” *Age 65*
	Social Support beyond family and friends (*n* = 37)	“Well, my doctor always tries to encourage me to lose a few pounds, but it’s nothing serious. He doesn’t really press me too much. He just says, in general, it’s better to lose a few. I try to listen to him because I’m at the point to lose 10 or 15. But now, maybe he’s saying maybe lose five. So, I have lost.” *Age 54*

## Discussion

Currently, limited research exists that qualitatively examines the desires of Black adults for the creation of weight loss interventions by learning about what supports or creates a barrier to diet and physical activity behaviors. This qualitative study investigated the barriers and facilitators to weight loss behaviors such as healthy diet and physical activity and the individual weight loss program preferences of Black adults. The study sample closely mirrored the demographic characteristics of the Black population in Connecticut. As of 2023, Black adults represented 13.1% of the state’s population. Among this demographic, 38.3% experienced obesity, 85.4% had attained a college degree, 29.8% were married, and 52.4% had never married [23–25]. Additionally, 63.1% of Black adults were employed, with a median annual household income of $32,000. Participants in the current study also represented from various states across the country. In 2024, 40% of Black adult men and 58% of Black adult women in the country were obese, with Black individuals comprising 14.4% of the country’s population, 17% residing in the Northeast, having a median income of $54,000, and 27% holding a college degree [26, 27]. Major themes that emerged from the interviews included preferences for weight loss interventions among both Black males and Black females, barriers to adopting healthy eating and physical activity, healthy living facilitators, and desire for social support. Centering on the weight loss intervention design for Black adults, the responses revealed some clear distinctions in the preferences of Black males and females. For Black females, the sub-themes generated were community and personalization while for Black males, programs focusing on personalized exercise and healthy eating for managing various health conditions emerged as major sub-themes for weight loss program creation.

Black males and females in the sample identified unique, sex-specific needs for weight loss with small areas of similar desires for weight loss program creation. Inclusion of community was highlighted by Black females. Specifically, desires for community, a source of support, as part of weight loss intervention design was raised. Black females in the sample discussed the importance of including pre-existing community programs in obesity treatment. This finding aligns with recommendations for obesity control and equity where provision of and support for existing community programs that seek to improve physical activity and nutrition is a key strategy [28, 29]. In comparison to the current study, qualitative research on the weight loss needs of adults from diverse racial backgrounds highlighted that a sense of community within weight loss programs helps address gaps in willpower arising from negative self-perceptions [30]. Consequently, it was recommended that municipalities implement strategies to effectively integrate multiple levels of support into weight loss programs [30]. This may look like obesity treatment alongside nutrition assistance programs that improve access to social and economic resources or treatment through community programs like Black Girls Run! that already focus on a weight-related behavior (physical activity) and provide social support to an engaged audience [14, 31]. Community-engaged efforts like the Racial and Ethnic Approaches to Community Health across the United States (REACH US) project that utilized participatory methods to generate multi-level, culturally and contextually relevant health interventions, reduced obesity prevalence across fourteen Black communities [32].

Males in this study wanted weight loss interventions designed specifically for them. Incorporating both eating and exercise was of equal importance to Black males as a means to reduce weight and address comorbidities like diabetes and hypertension. This finding is supported by the literature among older Black males, where diagnosis with a disease is noted as a motivator to participate in health research for better disease management and understanding [33]. Gender-specific weight control programs may be relevant as weight loss strategies differ between Black males and females [34, 35]. For example, Black male respondents to a community survey reported more regular exercise engagement than Black females and had less engagement with weight loss strategies and attempts [36]. Findings from other research representing adults from both genders suggest that structuring weight loss interventions to encourage participants to focus on and achieve success with one behavior change at a time, such as improving dietary intake, can significantly enhance self-efficacy, an important factor in behavior change [37, 38]. Across genders, personalization through culturally relevant dietary prescriptions and exercise was seen as necessary by Black adults in this qualitative study, which resonates with current weight control literature to address inequities. Still, gender-specific needs exist in weight-related behaviors like physical activity that indicate the relevance of race- and gender-specific weight control treatment as a future direction [36, 39].

Participants recognized several barriers to consuming healthy meals. Black females emphasized that their primary barriers to cooking and consumption of healthy foods were lack of time to procure and prepare nutritious meals and family obligations. Unlike the findings of this study, several qualitative studies examining barriers to healthy eating mentioned a lack of knowledge to be the major challenge to healthy eating and expressed uncertainty about what and how to cook healthy, in contrast to the current study where lack of time was identified as the primary barrier [40, 41]. Additionally, qualitative study findings in Black women indicated that women felt pressured to consume more, lacking support when attempting to eat healthy and reported that this created a hindrance to adopting healthy eating habits [41, 42].African American women, in a research study examining attitudes and perceptions of obesity and explored the preferences for weight loss interventions emphasized that their cultural background significantly influenced their dietary habits, often contributing to unhealthy eating patterns [43]. Still, they highlighted the importance of incorporating culturally relevant information about common foods when designing effective weight loss programs. Women in a qualitative study examining self-perceived barriers and facilitators to weight loss and maintenance among women from diverse racial backgrounds participants in weight loss interventions identified meal replacements as a significant barrier to consuming healthy foods during weight loss [44]. Participants reported that meal replacements were often unappealing due to their unpleasant taste, limited flavor variety, and lack of proper guidance on changing their dietary habits to support weight loss which made their experience tedious and monotonous [44]. On the other hand, lack of knowledge, skills, and a shortage of healthy recipes was identified as major barrier by males. To address this issue, future weight loss interventions should focus on integrating cooking methods, stressing the importance of portion control, and helping understand the nutritional value of foods [35]. Programs should also aim to incorporate a range of resources, including cooking classes, online tutorials of culturally relevant recipes, and community programs dedicated to healthy cooking [45, 46]. As Black males, in the current study, expressed a desire for recipes that could assist in managing health conditions such as hypertension, the use of the DASH (Dietary Approaches to Stop Hypertension) diet, which has been proven effective in controlling hypertension and supporting weight loss, may be beneficial and attractive [47, 48].

Black females and Black males identified distinct barriers to engaging in physical activity. Black females specifically identified challenges related to busy work schedules and caretaking responsibilities, leading to fatigue and tiredness, leaving little energy for exercise [14, 49].Previous studies have explored unique challenges Black women face regarding physical activity highlighting barriers such as hair care and cultural perceptions of body image [50, 51]. In a study by Tay, et al.(2023), environmental factors were specified by adults from both genders as major barriers to physical activity for weight loss [44]. For instance, lack of access to exercise facilities and pricey commercial gym memberships were indicated as two most prominent de-motivators to engaging in physical activity. In the context of physical activity, utilizing home-based programs through weight loss interventions, personalized to the level of resources available, may provide significant improvement and adherence to physical activity [52]. Black males noted a lack of motivation to maintain a physical routine. This finding is consistent with qualitative research findings that suggested Black males experience decreased motivation to participate in physical activity due to challenges and stress associated with fulfilling key gendered social roles as a worker, provider, father, and spouse/partner [31, 53]. Black male participants desired personalized exercise regimens for different age groups to enhance motivation and distributed the exercise schedule in small intervals throughout the week. When designing interventions for Black males, it is essential to incorporate exercises preferred by men and create a consistent routine to perform these exercises [12, 53, 54].

Black adult participants noted what supports or would be beneficial in their healthy eating and physical activity pursuits. A review of qualitative studies on adults of mixed racial backgrounds identified psychosocial factors like external accountability, supervision from health experts, and motivation—particularly among those with pre-existing chronic health conditions (e.g. type 2 diabetes, fatty liver disease, and cardiovascular disease)—as key facilitators for healthy eating, physical activity, and weight loss behaviors [44]. A distinctive finding of the current study findings is that participants of both genders emphasized the use of technological resources as a facilitator for promoting healthy eating and physical activity. Special reference was made to personal devices or smartphone apps for tracking weight, and diet, recording daily physical activity, and connecting to a health coach remotely for guidance and problem-solving. Despite the high usage of smartphones and smartphone-related tools and apps in Black adults (83%), few studies have used smartphone apps for tracking diet and physical activity and targeted this population [55–57]. This indicates that future interventions could use digital modalities for delivering weight loss programs. Considering the personalized weight loss program preference among both genders, future research in this area should aim to provide tailored messages and individualized intervention components [45–47]. These can comprise languages and terminologies that connect effectively with both Black males and Black females. It is likely essential to investigate the most effective ways to deliver personalized interventions through digital technology.

Female participants expressed a preference for seeking social support from social contacts primarily from friends and family. They also indicated a desire for guidance on healthy eating, particularly from healthcare professionals such as dieticians, valuing their expertise in this area. Findings from a research study by Tucker et al. (2022), show similar trends where Black women emphasized that the weight loss journey depends on an effective communication from the healthcare providers and feeling comfortable trusting the advice provided by them [58]. In line with a study by Bowie et al. (2018), Black female participants stressed the significance of learning culturally relevant diet and exercise information from healthcare providers and connecting with them remotely for problem-solving. Female participants expressed feeling most supported when engaging in healthy behaviors with family members [59]. Similarly, Jenkins et. al.(2017), found Black women highlighted the positive impact of having a family member as an exercise or walking partner, contributing to their physical activity goals and facilitating weight loss [52].

While social support for weight loss behaviors in Black males has been less studied, some research findings indicated that having a friend or peer with similar health goals provides encouragement and accountability for physical activity goals [60, 61]. This study identified that Black male participants expressed a need for friends and coworkers to participate in both dietary and physical activity behaviors. In a qualitative study, exploring perceived motivators and barriers in adopting healthy eating and physical activity behaviors, young males highlighted social and societal barriers to healthy eating, including peer influence, group dining norms and stereotypes associating healthy diets with a less masculine image [62]. In both Black female and male participants, social support has been recognized as a major factor for improving diet and physical activity and requires support from family members, friends, and coworkers. A qualitative study examining longitudinal relationships between sources of social support, social undermining for healthy eating and physical activity, and weight change among adults from diverse racial backgrounds found that family support had no significant impact on healthy eating behaviors [63]. Instead, participants highlighted that social support from friends and coworkers played a key role in encouraging a healthy diet. A study with Black women used qualitative thematic synthesis and an intersectionality framework to analyze existing research on barriers to and facilitators of physical activity [50]. The findings highlighted that key motivators include positive and encouraging social support, personal testimonials promoting physical activity, an active social environment, and observing neighbors engaging in exercise. Weight loss interventions should aim to incorporate social support subscales for both genders, enabling a deeper understanding of how the involvement of family and friends influences each aspect of diet and physical activity behaviors with Black adults [64, 65].

This study has both strengths and limitations. A notable strength lies in the inclusion of diverse Black male and female participants from various geographical locations. Another notable strength is the diversity encompassing the ancestry, socioeconomic statuses and educational backgrounds of the participants. Participants self-identified as African American, Afro-Caribbean (mostly Jamaican), and therefore reflected a variety of cultural heritage. The participants included Black adults from various socioeconomic and educational backgrounds. However, the sample was predominantly female (67%) potentially limiting their generalizability to a broader national sample. Another limitation is that the data collection occurred during the COVID-19 pandemic, potentially impacting participant’s responses due to the unique circumstances during that time which may have shifted their typical behavioral engagement to modify diet and physical activity. The participants, with an average age of 46.1 years, were mostly single (54%) and therefore the findings about weight loss program creation may not be generalizable to individuals who are older (65 years or more) or living with a partner. The current study captured the desires of both the sexes for a weight loss program, facilitators and barriers to modifying diet and physical activity. Though we acknowledge limitations in generalizability are present, the utilization of the qualitative approach allowed for a focused effort towards understanding the unique needs of Black adults for engagement in weight loss interventions for traditionally in-person and digital modalities. Future qualitative studies should aim toward addressing this identified gender imbalance to strengthen the transferability of findings.

### Implications for future research

The study findings indicate the unique weight loss preferences of Black adults with overweight or obesity. The results signify the importance of support from friends and family and the use of technological resources most beneficial to adopting weight loss behaviors. Female participants prioritized the need for a community and personalization for weight loss programs and expressed a desire to learn cooking skills and recipes to support culturally relevant meal preparation. While weight loss was the primary focus, male participants expressed a desire to learn about other health conditions, such as type 2 diabetes and hypertension and diets that could help manage these conditions. The findings hold relevance when designing weight loss interventions for including Black adults. Future research in this area should consider the weight loss preferences of Black adults across diverse ethnicities, considering individuals living with various chronic health conditions and utilizing a greater nationally representative sample.

## Data Availability

In order to safeguard participant privacy and comply with University of Connecticut’s Institutional Review Board regulation, the data analyzed in this study are not accessible to the public. Please reach out to Dr. Loneke Blackman Carr for any data related inquiries at loneke.blackman_carr@uconn.eduEthics approval and consent to participateAll study procedure was approved by the University of Connecticut’s Institutional Review Board (L20-0031). Each participant provided informed consent before joining the study. All study procedures were performed as per guidelines.

## Abbreviations/Acronyms

BMI: Body mass index
